# The photoswitchable ABAMIs for the future regulation of GWT1 in a spatiotemporal level

**DOI:** 10.1002/smo2.70053

**Published:** 2026-05-06

**Authors:** Qian Ding, Zongying Li, Zaiwei Xiao, Siting Zhang, Zimai Liu, Xiaoguang Shao, Zhong Li, Wen Fu, Xusheng Shao

**Affiliations:** ^1^ Shanghai Key Laboratory of Chemical Biology School of Pharmacy East China University of Science and Technology Shanghai China; ^2^ School of Pharmaceutical Sciences Shandong University Jinan Shandong China; ^3^ Agricultural Comprehensive Service Center of Xiaqiu Town Yantai Shandong China; ^4^ State Key Laboratory of Bioreactor Engineering East China University of Science and Technology Shanghai China; ^5^ State Key Laboratory of Green Pesticide Guizhou University Guiyang Guizhou China; ^6^ Shanghai Frontier Science Research Base of Optogenetic Techniques for Cell Metabolism School of Pharmacy East China University of Science and Technology Shanghai China; ^7^ Engineering Research Center of Pharmaceutical Process Chemistry Ministry of Education School of Pharmacy East China University of Science and Technology Shanghai China

**Keywords:** azobenzene‐aminopyridine, bioactivity, GWT1, pesticide discovery, photoswitchable

## Abstract

Photoswitchable bioactive molecules have attracted more attention and are widely used as photochromic ligands (PCLs) in both agrochemicals and pharmaceuticals. The glycosylphosphatidylinositol (GPI) acyltransferase GWT1 has been identified as a novel agricultural target of the 2‐aminopyridine fungicides. Herein, the GWT1‐targeted azobenzene‐aminopyridine derivatives (**ABAMIs**) were designed and synthesized to spatiotemporally regulate biological functions, which are promising tools in revealing the unclear antifungal mechanism. The maximal nine‐fold difference in activity was found between UV‐irradiated and unirradiated **ABAMI1** against *Botrytis cinerea* (*B*. *cinerea*). This, in principle of concept, could realize the regulation of GWT1 functions. Furthermore, a three‐fold bioactivity difference against *Sclerotinia sclerotiorum* (*S*. *sclerotiorum*) was observed between the *trans‐* and *cis*‐*p* **F**_**4**_ **ABAMI**s that were sensitive to visible light. The **ABAMIs** were successfully developed as PCLs that were allowed to modulate fungicidal activity by light, providing innovative and powerful tools for understanding functional GWT1 and opening an avenue to the GWT1‐targeted pesticide discovery.

## Graphical abstract

The GWT1‐targeted azobenzene‐aminopyridine derivatives (**ABAMIs**), sensitive to the light at wavelengths from 365 to 520 nm, are separately developed as photochromic ligands to regulate bioactivity as well as the GWT1 functions. These molecules are smart tools in photopharmacological studies, giving an innovative method for understanding functional GWT1 and pesticide discovery.

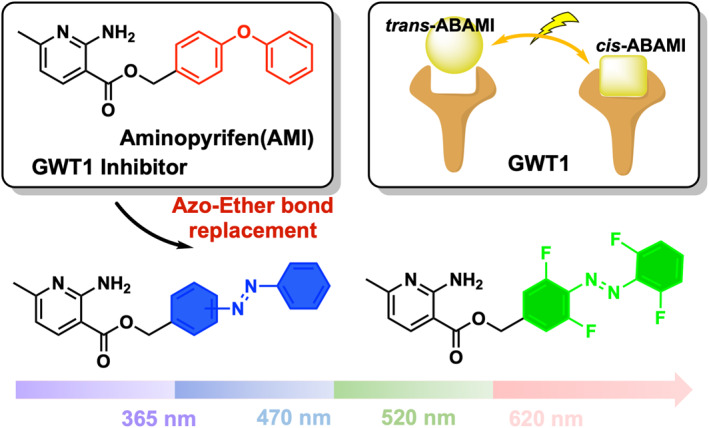

## 1 INTRODUCTION

Photopharmacology, a rapidly developing photonics‐based field of research, provides an effective strategy for precise light‐driven manipulation of biological functions.[^1^, ^2^, ^3^] This technology can help us understand the interaction mechanisms between the targets and photochromic ligands.[^4^, ^5^] Recent advances have further highlighted the potential of photopharmacology in both basic research and precision agriculture, with emerging concepts and tools being continuously reported.[^6^, ^7^] Azobenzene is one of the most described photoswitches used to design photoswitchable molecules.[^8^, ^9^] It existed in two distinct isomers and could undergo *trans*/*cis* reversible photoisomerization, giving rise to robust changes in the end‐to‐end distance and geometry of the molecule. Therefore, azobenzene can be an effective tool to modulate biological activities by regulating the affinity of a ligand to the receptor under the condition of light.[^1^, ^10^, ^11^] The rational design of azobenzene‐based photoswitches has been extensively explored to achieve efficient photoisomerization with minimal fatigue, and their applications in photopharmacology have been systematically reviewed.^[12]^ Azobenzene also has the advantages of readily synthesis, design flexibility, minimal photobleaching, and rapid responsivity under bio‐related conditions.[^13^, ^14^, ^15^, ^16^] In addition, the visible light‐sensitive azobenzene derivatives, designed by incorporating fluorine, chloro, or methoxyl into the *ortho* positions of the azo bond, have been developed to solve the limitation of ultraviolet in toxicity and permeability.[^17^, ^18^] The development of visible‐light‐responsive azobenzene derivatives has further expanded the applicability of photopharmacology in complex biological systems, enabling spatiotemporal control with reduced phototoxicity.[^19^, ^20^]

Aminopyrifen, discovered by Agro‐Kanesho, is a broad‐spectrum fungicide with a novel mode of action.^[21]^ It is not cross‐resistance to benzimidazole, anilinopyrimidine, dicarboximide and other commercialized fungicides,^[22]^ and shows excellent fungicidal activity against various plant diseases including gray mold, sclerotinia rot, powdery mildew, wheat head blight and anthracnose.^[23]^ As the first antifungal agent against GWT1 protein,^[24]^ it is an opportunity and great challenge to develop the GWT1‐targeted photochoromic ligands into good model molecules for studying the function of GPI‐anchored wall transfer proteins and the mechanism underlying the maintenance of fungal cell wall integrity.^[22]^

**SCHEME 1:**
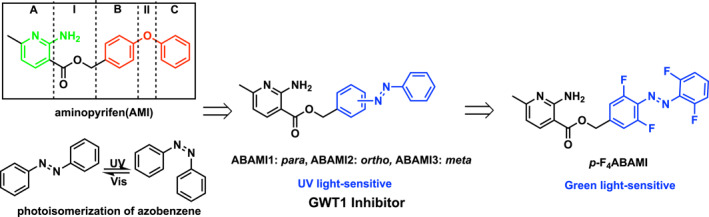
Design of the target compounds.

As we know, the structures of the reported GWT1 inhibitors generally bear three regions, A, B, and C, which are the aryl rings connected by two linkers, I and II (Scheme 1). Ring A is 2‐aminopyridine, a significant functional fragment for retaining fungicidal activity. In this work, we replaced link II with an azo bond and prepared a series of photoswitchable **ABAMIs**. **ABAMI1**‐**3** and *p* **F**_**4**_ **ABAMI** were evaluated for their photophysicochemical properties, fungicidal activities and mycelia‐inhibiting mode.[^25^, ^26^] The external ultraviolet and visible light were advisable stimuli to convert the configurations of the **ABAMIs**. It may provide an interesting and powerful tool for the spatiotemporal regulation of bioactivities and other interactional mechanisms, especially for the understanding of the GWT1 proteins.

## 2 MATERIAL AND METHOD

### 2.1 Instruments and chemicals

^1^H nuclear magnetic resonance (^1^H NMR) and ^13^C NMR data were acquired on a Brucker Ultra Shield (400 MHz) with DMSO‐*d*_6_ or CDCl_3_ as the solvent. TMS was used as the internal standard, and chemical shifts were reported as a *δ* (ppm) value. The melting point was determined using a Melting Point MP‐420. High‐resolution mass spectrometry (HRMS) data were obtained on a GCT Premier instrument under electron impact ionization condition. The UV‐Vis spectra were recorded with a Shimadzu UV‐1900 UV‐Vis Spectrophotometer. The cis/trans ratio was recorded with SHIMADZU Nexera LC‐30A. All reagents were of analytical or chemical purity, and solvents were subjected to drying pretreatment prior to reactions when necessary.

### 2.2 Photophysicochemical properties

*Test of UV‐Vis spectra*. A 2 × 10^−5^ M solution of the target compound was prepared with acetonitrile as the solvent and stored in the dark at room temperature for 24 h. The prepared solution was transferred in a 1 × 1 cm cuvette and irradiated with 365 nm or 520 nm light. Meanwhile, its absorbance in the range of 500‐200 nm was recorded until the absorbance had no changes.^[27]^

*Test of cis/trans ratio*. The solution prepared for UV‐Vis spectra assays was used to determine the *cis/trans* ratio by Ultra Performance Liquid Chromatography. Then, the solution was irradiated with 365 nm or 520 nm light, after which the *cis/trans* ratio was recorded.

*Test of half‐life*. The prepared solution for UV‐Vis spectra was used to determine the half‐life time by UV‐Vis spectrophotometer. Its absorbance at *λ*_*trans‐max*_ was recorded. Then, the solution was fully irradiated with 365 nm or 520 nm light to maximize the conversion of the *trans* configuration to the *cis* configuration. Subsequently, the solution was stored in the dark, and its absorbance was recorded at intervals.^[28]^

### 2.3 Fungicidal activity and illumination conditions for bioassays

The test method was based on that described in the literature^[29]^ with minor modifications. For bioactivity evaluation, the target compounds were first dissolved in DMSO, and then diluted with 0.5% Tween 80 aqueous solution to achieve the desired concentrations. The solutions were placed in a light‐proof box and irradiated with 365 nm (for ABAMI1–3) or 520 nm (for *p*‐F_4_ABAMI) light at an intensity of 2.5 mW/cm^2^ and 3.0 mW/cm^2^, respectively, with a distance of 5 cm from the light source. Irradiation was continued for at least 3 hours to ensure maximal photoisomerization. After irradiation, the samples were immediately incorporated into the PDA medium for antifungal assays.

The 9‐cm petri plates were each loaded with 15 mL of sterile potato dextrose agar (PDA) medium, supplemented with a gradient of concentrations of target compounds (pre‐ and post‐irradiation) and Aminopyrifen. Mycelial cakes (5 mm in diameter) from the margin of 2‐day‐old colonies were transferred into PDA plates. Following incubation at 25°C in the dark for 2–3 days, the mean colony diameter was measured and the EC_50_ values were calculated using SPSS software. Three biological replicates were used for each treatment.

### 2.4 Micromorphology analysis

Scanning electron microscope (SEM) was employed to observe the effects of **ABAMI1** on the mycelial micromorphology of *Botrytis cinerea* and *Sclerotinia sclerotiorum* as well as the effect of *p*‐**F**_**4**_ **ABAMI** on the mycelial micromorphology of *S*. *sclerotiorum*. The sample preparation and SEM observation procedures were carried out according to the method described in our previous work.

### 2.5 Molecular docking

The cryo‐electron microscopy structure of the GWT‐1 protein in complex with manogepix was obtained from the PDB database (ID: 8XIK). Molecular docking studies were conducted using the Schrödinger 14.0 software package. The protein structure was first subjected to preprocessing, including removal of water molecules, completion of missing residues, optimization of the hydrogen bonding network, and energy minimization. The docking grid was defined centered on the ligand from the original crystal structure, with grid dimensions set to fully encompass the binding pocket. The ligand molecules (ABAMIs) underwent energy minimization under the OPLS4 force field to obtain stable conformations. Subsequently, docking was performed using the Glide module, where the optimized ligands were redocked into the binding pocket of the protein, generating 10 representative binding conformations. To evaluate the specificity and similarity of the binding modes, the positive fungicide aminopyrifen was used as a control. By comparing the interactions of each docking conformation with those of aminopyrifen at the binding site with key amino acid residues (e.g., hydrogen bonds, hydrophobic interactions), binding conformations exhibiting similar interaction patterns to aminopyrifen were selected for analysis.

## 3 RESULTS AND DISCUSSIONS

### 3.1 Molecular design and synthesis

**SCHEME 2:**
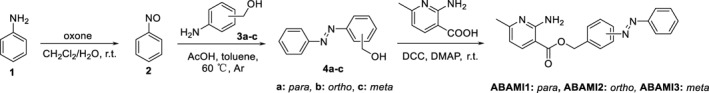
The synthetic route of the UV light‐sensitive compounds.

Based on the known structure‐activity relationships of GWT1 inhibitors against the fungi, the ether bond at linker II was substituted with an azo bond, leading to the successful synthesis of three novel azobenzene compounds with substitution at *ortho‐*, *para‐*, and *meta‐*positions. The molecular design strategy is illustrated in Scheme 1.^[30]^ **ABAMI1**‐**3** were synthesized via a three‐step reaction sequence with aniline and 4‐aminobenzenemethanol serving as the starting material. The oxidation of aniline **1** was oxidized by oxone to yield nitrosobenzene **2**, which was then subjected to the Mills reaction with aminobenzenemethanol **3a**‐**c** to produce phenylazobenzyl alcohol **4a**‐**c**. Finally, the esterification condensation of phenylazobenzyl alcohol **4a**‐**c** with 2‐amino nicotinic acid furnished the target compounds **ABAMI1**‐**3** (Scheme 2).

**SCHEME 3:**
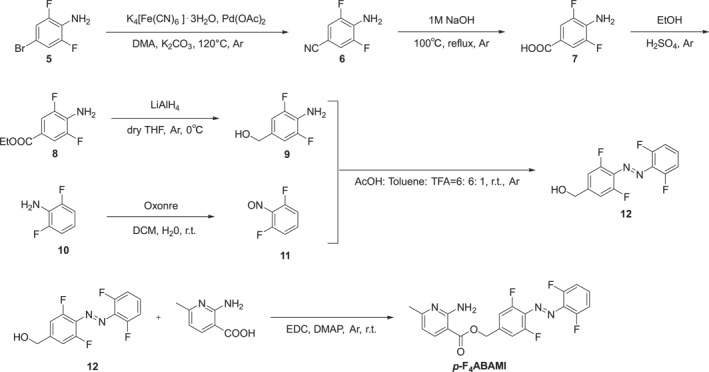
The synthetic route of the green light‐sensitive compound.

Fluorine atoms were incorporated into the azobenzene moiety to obtain compound ***p*‐F**_**4**_ **ABAMI**. The molecular design is illustrated in Scheme 1. Given the synthetic challenge associated with 4‐amino‐3,5‐difluorobenzyl alcohol **9**, multiple reaction steps were required for its synthesis. However, the subsequent synthetic pathway was analogous to that of the reaction with azo benzene benzyl alcohol. Ultimately, despite the relatively low yield, the target compound ***p*‐F**_**4**_ **ABAMI** was successfully synthesized (Scheme 3).

### 3.2 Photophysicochemical properties

The photoisomerization properties of the target compounds were analyzed using a UV‐Vis spectrometer and high‐performance liquid chromatography to evaluate their suitability for subsequent biological assays, with the results summarized in Table 1. All compounds exhibited typical azobenzene absorption spectrum and photoswitching behavior. It should be noted that these photoisomerization properties were characterized in acetonitrile as a standardized solvent. Although the intracellular or mycelial microenvironment may influence the photoisomerization efficiency in vivo, the pre‐irradiation protocol employed in bioassays was designed to achieve maximal conversion of the trans isomer to the cis isomer under controlled conditions, which is comparable to the photoisomerization behavior observed in acetonitrile. Future studies may further investigate the impact of biological environment on photoisomerization efficiency.

**TABLE 1:** Maximum absorbance wavelength (λmax, nm), the ratio of trans‐ and cis‐isomers and the rate of the thermal relaxation of **ABAMI1**‐**3**.

Compound	*λ*_max π–π*_ (*trans*, nm)	Non‐irradiated (*trans*:*cis*)	*λ*_max n–π*_ (*trans*, nm)	Irradiated (*trans*:*cis*)	t _1/2_ (h)
ABAMI1	328	100:0	433	46:54	135.3
ABAMI2	325	93:7	435	38:62	427.5
ABAMI3	324	100:0	437	47:53	446.5
*p*‐F_4_ABAMI	326	95:5	450	22:78	6837.5

**FIGURE 1:**
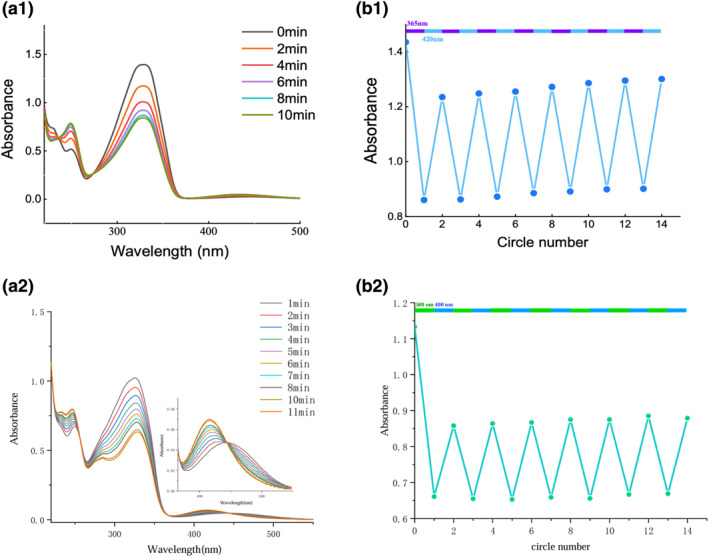
UV‐Vis spectra of **ABAMI1** and ***p*‐F4ABAMI** before, during and after irradiation (a1, a2) and fatigue resistance of **ABAMI1** and ***p*‐F**_**4**_ **ABAMI** (b1, b2).

The changes in the UV‐Vis spectra indicated that *trans* **‐ABAMI1** and *trans*‐*p*‐**F**_**4**_ **ABAMI** were gradually converted to the *cis* configurations following 10 min of UV (365 nm) light and a 11 min of green (520 nm) light, respectively. The maximum absorption wavelength for the π–π* transition of the *trans*‐**ABAMI1** was 328 nm, while that of the n–π* transition of *cis*‐**ABAMI1** was 433 nm (Figure 1a). As expected, the maximum absorption wavelength for the n–π* transition of *cis*‐*p*‐**F**_**4**_ **ABAMI** was shifted to 450 nm. Upon repeated UV‐blue light illumination, the isomerization could be triggered for multiple cycles without significant fatigue (Figure 1b). The half‐life of the *cis*‐isomers to *trans*‐isomers varied from 5 to 285 days, falling within the appropriate time window for biological assays. All **ABAMIs** exhibited photoisomerization efficiency of 53%–73% for *trans* to *cis* configuration.

### 3.3 Fungicidal activity

**FIGURE 2:**
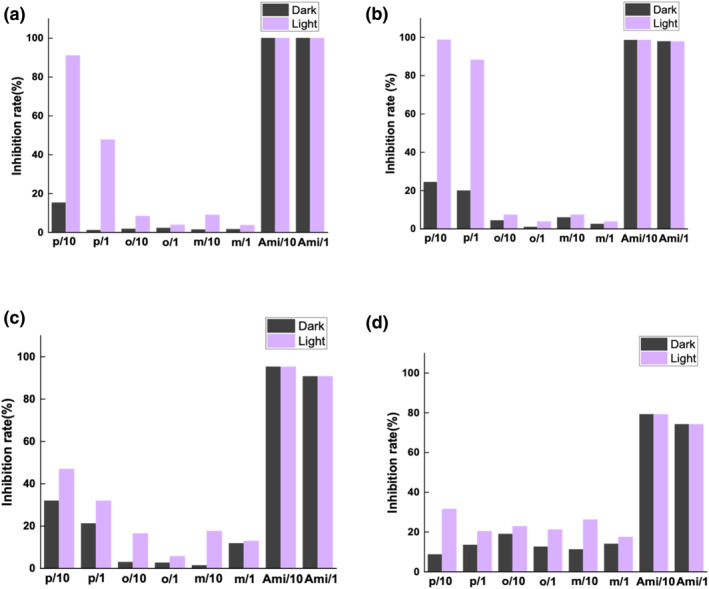
Fungicidal activities of the target compounds. Here, *p*, *o*, *m* means **ABAMI1**, **ABAMI2**, **ABAMI3**; Ami means Aminopyrifen; 10 and 1 represent the concentration, and the unit is *μ*g·mL^−1^. (a) Inhibition rate of mycelial growth by *Botrytis cinerea* isolates treated with compounds before and after irradiation. (b) Inhibition rate of mycelial growth by *Sclerotinia sclerotiorum*. (c) Inhibition rate of mycelial growth by *Fusarium graminearum*. (d) Inhibition rate of mycelial growth by *R*. *Solani*.

**ABAMI1**‐**3** and Aminopyrifen were assessed for their fungicidal activities against *Botrytis cinerea*, *Sclerotinia sclerotiorum*, *Fusarium graminearum* (*F*. *graminearum*), and *Rhizoctonia solani* (*R*. *Solani*). As shown in Figure 2, irradiated **ABAMI1** showed a certain inhibition rate to the four fungi and the inhibition activity exhibited a big difference before and after irradiation. At 10 *μ*g·mL^−1^, irradiated **ABAMI1** showed an inhibition rate of over 90% against *B*. *cinerea* and *S*. *sclerotiorum*; besides, even at 1 *μ*g·mL^−1^, irradiated **ABAMI1** reached an 88% inhibition rate against *S*. *sclerotiorum*. Non‐irradiated **ABAMI1** only exhibited weak fungicidal activity. The biological results indicated that **ABAMI1** can be photoactivated, allowing for optical regulation of its fungicidal activity. However, the fungicidal activity of **ABAMI1** was inferior to that of Aminopyrifen, which could be attributed to two reasons: (I) The conversion rate of **ABAMI1** to its cis configuration following light irradiation was only approximately 50%, which compromised its fungicidal activity; (II) The diphenyl ether has a specifical binding affinity for the target, and replacement of this moiety with an azo bond impaired the ligand‐receptor interaction. To further explore this, molecular docking studies were performed revealing that the replacement of the diphenyl ether moiety with an azo bond led to a subtle but distinct change in the binding mode (see Section 3.5). Significantly, regardless of irradiation and after irradiation, **ABMAI2** and **ABMAI3** showed negligible activity against the four fungi. The results indicated that the installation position on the phenyl position has a significant influence on the activity. Due to the obvious difference in fungicidal activity between different structures, further researches can be conducted on the structure‐activity relationships of aminopyrifen analogs.

**FIGURE 3:**
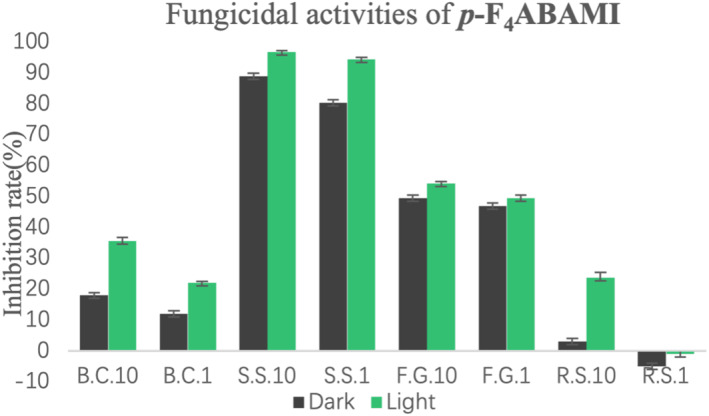
Fungicidal activity before and after green irradiation.

The fungicidal activities of the target compound *p‐* **F**_**4**_ **ABAMI** were evaluated against the four strains (Figure 3). The results indicated that, in comparison with compound *p*‐**ABAMI**, the bactericidal activities of compound *p‐* **F**_**4**_ **ABAMI** changed significantly, with an overall reduction in the activity difference between pre‐ and post‐irradiation. Specifically, post‐irradiation, the inhibition rates of *p‐* **F**_**4**_ **ABAMI** against *B*. *cinerea* and *R*. *Solani* decreased. In contrast, post‐irradiation, the antifungal activity of *p‐* **F**_**4**_ **ABAMI** against *F*. *graminearum* was enhanced, despite the pre‐ and post‐irradiation activity difference being reduced to 1.1‐fold. Meanwhile, the antifungal activity of *p‐* **F**_**4**_ **ABAMI** against *S*. *sclerotiorum* was increased by 3.9‐fold post‐irradiation. The pre‐irradiation inhibition rate was increased by more than 10‐fold, while the pre‐ and post‐irradiation rates were also significantly narrowed.

**FIGURE 4:**
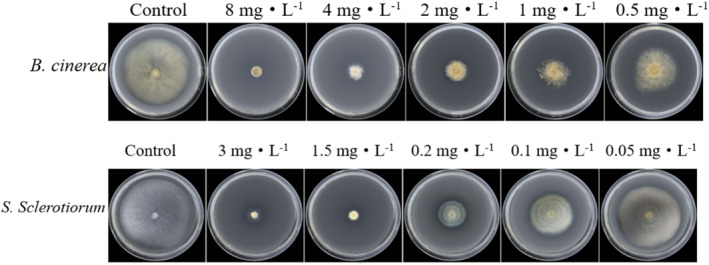
The antifungal efficacy of *cis*‐**ABAMI1**.

Based on the aforementioned assay results, the mycelial growth inhibition rate of irradiated **ABAMI1** against *Botrytis cinerea* and *Sclerotinia sclerotiorum* at different concentrations was further investigated, and EC_50_ values of irradiated **ABAMI1** were calculated by SPSS 16.0. The mycelial growth inhibition rate showed a dose‐dependent relationship with the concentration of the target compound (Figure 4). The higher the concentration of **ABAMI1**, the slower the mycelial growth of *B*. *cinerea* and *S*. *sclerotiorum*, with corresponding EC_50_ values of 0.926 *μ*g·mL^−1^ and 0.164 *μ*g·mL^−1^, respectively. Mycelium of *B*. *cinerea* and *S*. *sclerotiorum* was uniformly distributed along the surface of culture media without **ABAMI1**, and the colony margins were circular and regular. In contrast, when **ABAMI1** was added to the plates, the growth of mycelium was significantly inhibited. The colony margins were irregular, concave‐convex, and less smooth than those of the non‐treated group.

**FIGURE 5:**
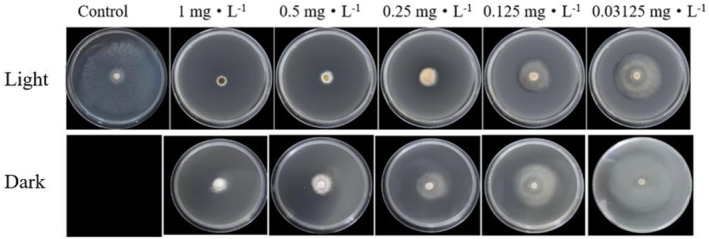
Colony growth chart of compound *p‐*F_4_ABAMI before and after green light.

Based on the initial activity screening, the EC_50_ values of the target compound against *Sclerotinia sclerotiorum* were tested under conditions of green light exposure (Figure 5). The EC_50_ value determined following green light exposure was 0.042 mg·L^−1^, in contrast to a value of 0.124 mg·L^−1^ detected in the dark. The difference in antifungal activity between light‐exposed and non‐light‐exposed conditions was 2.95‐fold, a reduction relative to the 4.25‐fold difference observed for the non‐fluorinated compound. As can be seen from the colony growth status in the figure, at the same concentration, the colony growth diameter of the light‐exposed group was smaller than that of the non‐light‐exposed group. At concentrations of 1 mg·L^−1^ and higher, the growth of *S*. *sclerotiorum* colonies was completely inhibited.

### 3.4 Morphology observation

**FIGURE 6:**
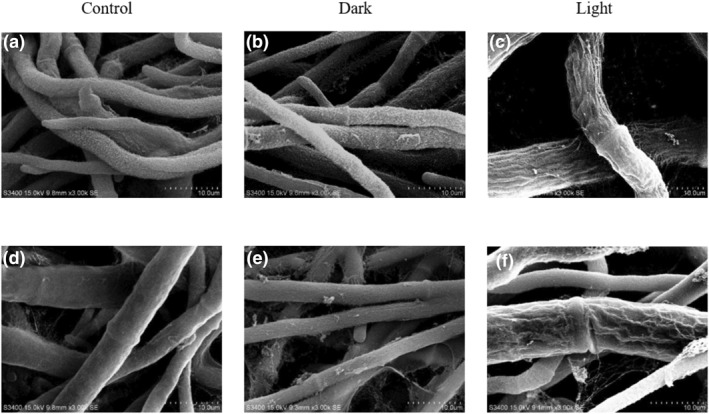
(a–c) Effect of **ABAMI1** on mycelia morphology of *Botrytis cinerea* after incubation for 3 d on PDA plate. The concentrations of **ABAMI1** were 1.0 *μ*g·mL^−1^ before and after light (b, c). (d–f) Effect of **ABAMI1** on the mycelia morphology of *S*. *Sclerotiorum* after incubation for 2 d on PDA plate. The concentrations of **ABAMI1** were 0.1 *μ*g·mL^−1^ before and after light (e, f).

The morphological characteristics of *Botrytis cinerea* and *Sclerotinia sclerotiorum* treated with **ABAMI1** were observed via Scanning electron microscope (SEM) (Figure 6). The control group exhibited normal linearly‐shaped mycelia with smooth surfaces and fine structures (Figure 6a,d), whereas the surface roughness and wrinkles of the mycelium treated with dark **ABAMI1** (Figure 6b,e). Following treatment with light‐exposed **ABAMI1**, the mycelia showed obvious shrinkage, adhesion and collapse (Figure 6c,f).

**FIGURE 7:**
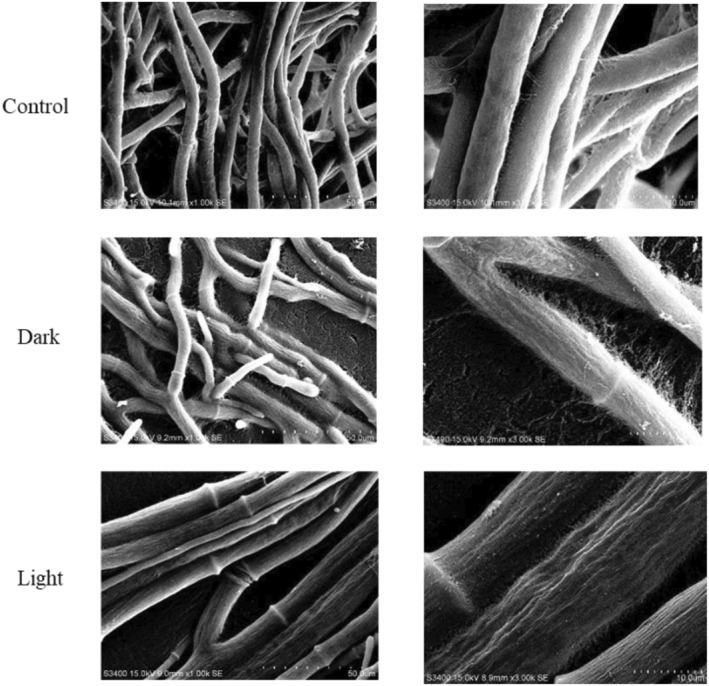
Effect of *p*‐**F**_**4**_ **ABAMI** on the mycelia morphology of *S*. *Sclerotiorum* after incubation for 50 h on PDA plate. The concentrations of *p*‐**F**_**4**_ **ABAMI** were 0.0625 *μ*g·mL^−1^ before and after light.

The morphological characteristics of *S*. *Sclerotiorum* following treatment with *p‐*F_4_ABAMI was observed using SEM (Figure 7). The blank control of *S*. *Sclerotiorum* exhibited the best growth condition. At 1000× magnification, abundant hyphae were observed with relatively regular growth patterns. At 3000× magnification, the hyphae presented intact morphologies with plump, smooth surfaces. By contrast, the hyphae growth status under dark conditions was inferior to that of the blank control. The hyphae were no longer distributed as regularly as those in the blank control, and slight wrinkles were observed on the hyphal surfaces, suggesting that the target compound had exerted a negative impact on the growth of *S*. *Sclerotiorum* under dark conditions. Following green light irradiation, the number of *S*. *Sclerotiorum* hyphae was decreased in the low‐magnification field of view. At 3000× magnification, more prominent wrinkles and shrinkage were observed relative to the dark‐treated group.

### 3.5 Molecular docking analysis

**FIGURE 8:**
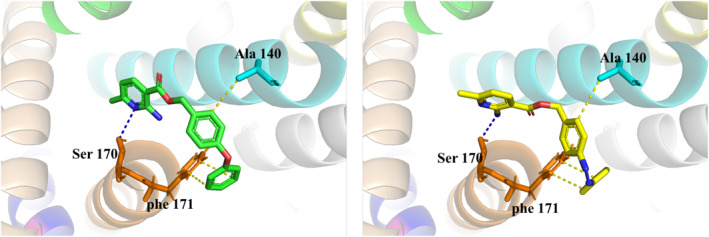
Molecular docking of Aminopyrifen and *cis*‐**ABAMI1** with the cryo‐EM structure of GWT1 (PDB: 8XIK).

To gain mechanistic insight into the activity differences between the cis and trans isomers and between the ABAMI compounds and aminopyrifen, molecular docking studies were performed using the cryo‐EM structure of GWT1 (PDB: 8XIK). The docking results (Figure 8) revealed that the cis isomer of **ABAMI1** was successfully docked into the binding pocket of GWT1 and exhibited a binding mode similar to that of aminopyrifen. In contrast, the trans isomer failed to dock into the active site, suggesting that the cis configuration is essential for effective binding. This observation provides computational support for the hypothesis that the light‐induced increase in antifungal activity is attributable to the formation of the cis isomer with improved binding affinity to GWT1.

Further comparison between aminopyrifen and the ABAMI analogs showed that the replacement of the diphenyl ether moiety with an azo bond led to a subtle but distinct change in the binding mode. Aminopyrifen formed a hydrogen bond with Ser170A via the nitrogen atom of its pyridine ring, whereas the ABAMI analogs interacted with the same residue through the nitrogen atom of the amino group. Both compounds shared common features in forming hydrophobic interactions with Ala140 and Phe171. These differences in hydrogen bonding patterns may partially account for the slightly‐reduced antifungal activity of ABAMI1 compared to aminopyrifen. Together, these docking results offer mechanistic insights into the photoswitchable activity of ABAMIs and support the role of GWT1 as the molecular target.

## 4 CONCLUSIONS

In summary, three azobenzene derivatives and one *tetra*‐*o*‐fluoroazobenzene derivative were designed and synthesized, and their fungicidal activities against four phytopathogenic fungi were evaluated. Irradiated **ABAMI1** exhibited satisfactory fungicidal activity against *Botrytis cinerea* and *Sclerotinia sclerotiorum*, with EC_50_ values of 0.926 *μ*g·mL^−1^ and 0.164 *μ*g·mL^−1^, respectively. Unirradiated **ABAMI1**, along with the *ortho‐* and *meta‐*analogs, showed almost complete loss of fungicidal activities. Compound **ABAMI1** not only exhibited potent inhibitory activity against *B*. *cinerea* and *S*. *sclerotiorum* but also presented a promising *trans*/*cis* activity difference, indicating its potential as an effective photoactivated fungicide that merits further investigation. *P*‐**F**_**4**_ **ABAMI** is capable of undergoing the trans/cis isomerization without causing additional damage to bacteria, demonstrating better thermal stability and a higher rate of photoisomerization. Against *S*. *sclerotiorum*, the EC_50_ value following green light irradiation is 0.042 mg·L^−1^, and the difference in antifungal activity before and after light exposure is 2.95 fold. Overall, the replacement of the diphenyl ether part of Aminopyrifen with para‐substituted azobenzene or tetra‐o‐fluoroazobenzene showed equal fungicidal activity as Aminopyrifen and possessed good photoswitchable properties. As we know, this is the first report on photoresponsive Aminopyrifen fungicide so far. Molecular docking studies further supported that the cis isomer is the active configuration for binding to GWT1 and the azo substitution alters the hydrogen‐bonding pattern, providing a mechanistic basis for the observed activity differences. Aminopyridines containing a steric configuration similar to *cis*‐*para*‐azobenzene or diphenyl ether may act on GWT1 protein, so **ABAMI1 and** *p‐* **F**_**4**_ **ABAMI** may help study the function of GPI‐anchored proteins.

## CONFLICT OF INTEREST STATEMENT

The authors declare no conflicts of interest.

## ETHICS STATEMENT

We hereby declare that no animal or human subjects were involved in this study.

## Supporting information

Supporting Information S1

## Data Availability

The data that support the findings of this study are available from the corresponding author upon reasonable request.
