# Supplementary material for: The photoswitchable ABAMIs for the future regulation of GWT1 in a spatiotemporal level
Source: Smart Mol. 2026 May 6:e70053. Online ahead of print. doi: 10.1002/smo2.70053 (PMC13398718; doi:10.1002/smo2.70053)
Supplement: Supplementary file 1 — Supporting Information S1 [file SMO2-9999-0-s001.docx]

**Supporting Information**

**The Photoswitchable ABAMIs for the Future Regulation of GWT1 in a Spatiotemporal Level**

Qian Ding^1#^, Zongying Li^5#^, Zaiwei Xiao^1#^, Siting Zhang^1^, Zimai Liu^6^, Xiaoguang Shao^6^, Zhong Li^1,2^, Wen Fu^1,7*^, Xusheng Shao^1,2,3,4*^

^1^*Shanghai Key Laboratory of Chemical Biology, School of Pharmacy, East China University of Science and Technology, Shanghai 200237, China*

^2^*State Key Laboratory of Bioreactor Engineering, East China University of Science and Technology, Shanghai 200237, China*

^3^*Shanghai Frontier Science Research Base of Optogenetic Techniques for Cell Metabolism, School of Pharmacy, East China University of Science and Technology, Shanghai 200237, China*

^4^*Engineering Research Center of Pharmaceutical Process Chemistry, Ministry of Education, School of Pharmacy, East China University of Science and Technology, Shanghai 200237, China*

^5^*School of Pharmaceutical Sciences, Shandong University, Jinan 250000, Shandong China*

^6^ *Agricultural Comprehensive Service Center of Xiaqiu Town, Yantai 261433, Shandong China*

^7^*National Key Laboratory of Green Pesticide, Guizhou University, Guiyang 550025, Guizhou China*

^#^ Qian Ding, Zongying Li and Zaiwei Xiao contribute equally in this work.

*Corresponding authors:

Wen Fu, E-mail: fu_wen@ecust.edu.cn

Xusheng Shao, E-mail: shaoxusheng@ecust.edu.cn

**Table of contents**

1. Materials and General Methods
2. General Synthetic Procedure for the Target Compounds **ABAMIs**
3. Copies of NMR Spectra of Compounds
4. References
5. **General Synthetic Procedure for the Target Compounds ABAMIs**

*Synthesis of nitrosobenzene* ***2****.* A biphasic mixture of aniline (0.93 g, 10 mmol), Oxone® (6.15 g, 10 mmol), DCM (30 mL) and water (60 mL) was stirred at 25 ^o^C for 30 min until TLC indicated full conversion of the aniline. The mixture was extracted with dichloromethane (3×30 mL). The organic layer was dried with MgSO_4_ and the solvent evaporated in vacuo. The crude material nitrosobenzene **2** as a black-green oil was directly used in the next step without further purification or characterization.

*Synthesis of (E)-(4-(phenyldiazenyl)phenyl)methanol* ***4a****.* The nitrosobenzene **2** (0.32 g, 3.00 mmol) and p-aminobenzoic acid (0.24 g, 2.00 mmol) were dissolved in a mixture of toluene (12 ml) and glacial acetic acid (1.20 g, 20.00 mmol). The reaction system was protected by argon and left under stirring for 36 hours at 60 °C. Reaction progress was controlled by silica gel TLC to monitor the disappearance of the starting material **3a** (eluent: (petroleum ether)/(ethyl acetate), 5:1). Next, the mixture was poured into sat. aq. NaHCO_3_ (3×20 mL) and extracted with dichloromethane (2 🞪 30 mL). The combined organic layers were washed with water (2 🞪 30 mL) and brine (30 mL) respectively, dried over anhydrous Na_2_SO_4_. Purification by column chromatography, PE/EA=6:1 (v/v). The solvent was evaporated in vacuo yielding a yellow solid (0.217 g, yield 51.2%). ^1^H NMR (400 MHz, CDCl_3_): δ 7.92 (d, *J* = 8.1 Hz, 4H), 7.58-7.43 (m, 5H), 4.78 (s, 2H), 1.99 (d, *J* = 42.9 Hz, 1H) ppm.

*Synthesis of* ***ABAMI1****.*A dried flask was charged with 2-amino-6-methyl nicotinic acid (0.188 g, 1.24 mmol), 4-phenylazobenzyl alcohol **4a** (0.132 g, 0.62 mmol), and 4-dimethylaminopyridine (0.013 g, 0.1 mmol). The reaction system was protected by argon, and dry dichloromethane (10 mL) was added by syringe. DCC dissolved in DCM was injected into the flask slowly and the reaction was stirred overnight. After the reaction was complete (TLC), EtOAc (30 mL) was added and the organic layer was washed consecutively with H_2_O (3 x 20 mL) and brine (30 mL), dried with Na_2_SO_4_ and removal of the volatiles in vacuo. For spectroscopic characterization, the product was filtered through a small plug of silica using PE/EA (4:1) as eluent. After concentrating to dryness, ABAMI1 (0.103 g, yield 47.8%) was obtained as a yellow solid.

^1^H NMR (400 MHz, CDCl_3_): δ 8.08 (d, *J* = 8.0 Hz, 1H), 7.98-7.89 (m, 4H), 7.61-7.47 (m, 5H), 6.50 (d, *J* = 8.0 Hz, 1H), 6.81-6.27 (s, 2H), 5.38 (s, 2H), 2.41 (s, 3H) ppm.^13^C NMR (101 MHz, CDCl_3_): δ166.81, 163.86, 159.25, 152.62, 152.46, 140.33, 138.89, 131.16, 129.12, 128.65, 123.12, 122.91, 112.65, 103.13, 65.79, 24.74 ppm. HRMS: m/z calcd for C_20_H_18_N_4_O_2_ [M]^+^ 346.1430; Found 346.1433.

The derivatives (**ABAMI2**, **ABAMI3**) could be synthesized by the method similar to that described in the synthesis of **ABAMI1**.

*Data for* ***4b****:* yellow solid; yield: 91.9%; ^1^H NMR (400 MHz, CDCl_3_) δ 7.85-7.80 (m, 2H), 7.75 (dd, *J* = 7.7, 1.5 Hz, 1H), 7.49-7.33 (m, 6H), 4.97 (s, 2H) ppm.

*Data for* ***ABAMI2****:* yellow solid; yield: 70.0%. ^1^H NMR (400 MHz, CDCl_3_) δ 8.02 (d, *J* = 7.9 Hz, 1H), 7.95-7.89 (m, 2H), 7.76 (dd, *J* = 7.8, 1.4 Hz, 1H), 7.65-7.60 (m, 1H), 7.55-7.42 (m, 5H), 6.42 (d, *J* = 8.0 Hz, 1H), 6.87-5.96 (m, 2H), 5.91 (s, 2H), 2.37 (s, 3H) ppm. ^13^C NMR (101 MHz, DMSO-*d_6_*) δ 166.20, 163.44, 159.17, 152.13, 149.17, 139.78, 135.11, 131.71, 131.63, 129.45, 129.11, 122.70, 115.35, 111.59, 101.61, 61.91, 24.20 ppm. HRMS: m/z calcd for C_20_H_18_N_4_O_2_ [M+H]^+^ 347.1507; Found 347.1498.

*Data for* ***4c****:* dark yellow solid; yield: 74.3%; ^1^H NMR (400 MHz, CDCl_3_) δ 7.95-7.90 (m, 3H), 7.86 (dt, *J* = 7.3, 1.8 Hz, 1H), 7.55-7.46 (m, 5H), 4.80 (s, 2H) ppm.

*Data for* ***ABAMI3****:* yellow solid; yield: 89.8%. ^1^H NMR (400 MHz, CDCl_3_) δ 8.09 (d, *J* = 8.0 Hz, 1H), 7.97 (s, 1H), 7.96-7.88 (m, 3H), 7.57-7.46 (m, 5H), 6.89-5.96 (m, 2H), 6.49 (d, *J* = 8.0 Hz, 1H), 5.41 (s, 2H), 2.41 (d, *J* = 6.7 Hz, 3H) ppm. ^13^C NMR (101 MHz, DMSO-*d_6_*) δ 166.29, 163.54, 159.20, 151.97, 151.85, 139.93, 137.87, 131.65, 130.83, 129.71, 129.47, 122.57, 122.21, 121.81, 111.67, 101.55, 65.18, 24.24 ppm. HRMS: m/z calcd for C_20_H_18_N_4_O_2_ [M]^+^ 346.1430; Found 346.1428.

*Synthesis of 4-Amino-3,5-difluorobenzonitrile* **6***.* A dried flask was charged with 4-bromo-2,6-difluoroaniline 5 (6.240 g, 30 mmol), potassium hexacyanoferrate (6.985 g, 6.6 mmol), palladium acetate as a catalyst (0.675 g, 3.1 mmol), and potassium carbonate (4.146 g, 30 mmol). Anhydrous N,N-dimethylacetamide (200 mL), freshly distilled, was rapidly added as the solvent, and the reaction system was then protected by argon. After stirring the mixture at room temperature to ensure homogeneity, the reaction system was heated to 120 °C and maintained for approximately 18 hours. Upon completion of the reaction (monitored by TLC), the reaction mixture was cooled to room temperature and diluted with ethyl acetate (100 mL). The resulting solution was filtered to obtain the filtrate, which was subsequently washed with distilled water (2 × 50 mL) and aqueous ammonia (2 × 50 mL), followed by extraction with ethyl acetate. The combined organic layers were dried over anhydrous magnesium sulfate. The product was purified by silica gel column chromatography using a hexane/ethyl acetate (4:1) eluent. The second eluting compound was isolated as the target product. After concentration to dryness, 4-amino-3,5-difluorobenzonitrile **6** was obtained as an off-white solid (3.23 g, yield 69.8%).^1^H NMR (400 MHz, CDCl3) δ 7.19-7.09 (m, 2H), 4.27 (s, 2H).

*Synthesis of* 4-Amino-3,5-difluorobenzoic Amide **7.** A flask was charged with compound 6 (3.082 g, 20 mmol) and 1 M NaOH aqueous solution (100 mL). A condenser was attached, and the reaction system was protected by an argon atmosphere. The reaction mixture was heated to 100 °C and maintained at this temperature for approximately 14 hours until the reaction was complete. After the reaction, the mixture was cooled to room temperature and slowly acidified with 1 M HCl solution until a large amount of white solid precipitated. The mixture was then extracted with ethyl acetate (100 mL, in three portions). The combined organic layers were dried over anhydrous magnesium sulfate. After removing the solvent by rotary evaporation, the crude product was obtained as a brownish-gray solid(3.13 g ,90.46%).^1^H NMR (400 MHz, DMSO-d6) δ 12.68 (s, 1H, -COOH), 7.50 - 7.31 (m, 2H), 6.07 (s, 2H, -NH2).

*Synthesis of* 4-Amino-3,5-difluorobenzyl Alcohol **9**.A dried flask equipped with a septum was purged with argon to protect the reaction. Anhydrous tetrahydrofuran (THF, 60 mL) was added, and lithium aluminum hydride (LiAlH₄, 1.06 g, 28 mmol) was introduced in portions under ice bath cooling. Each portion was approximately 0.25 g. After each addition, the flask was sealed and stirred for 5-10 minutes before the next portion was added. Once all the LiAlH₄ was added, stirring was continued until the reaction mixture turned gray-green.Compound 8 (3.50 g, 18.80 mmol) dissolved in anhydrous THF was then slowly added dropwise to the reaction mixture. The reaction was carried out under ice bath cooling for 30 minutes and then warmed to room temperature to continue. Upon completion of the reaction (monitored by TLC), the reaction mixture was quenched in an ice bath. Small portions of water and NaOH solution were carefully added in sequence, followed by additional water. The mixture was stirred for 30 minutes until all the LiAlH₄ was consumed. The reaction mixture was then diluted with ethyl acetate (EA), filtered through diatomaceous earth, and the filter cake was washed with EA. The filtrate was concentrated, dried over anhydrous sodium sulfate, and purified by column chromatography using a heptane/ethyl acetate (2:1) solvent system. The product 4-amino-3,5-difluorobenzyl alcohol **9** was obtained as a light brown solid (2.72 g, yield 91.3%).^1^H NMR (400 MHz, DMSO-d₆) δ 6.97-6.70 (m, 2H), 5.12 (t, *J* = 5.8 Hz, 1H, OH), 5.02 (s, 2H, NH₂), 4.33 (d, *J* = 5.8 Hz, 2H). ^19^F NMR (376 MHz, DMSO-*d*₆) δ -131.38 – -131.73 (m).

*Synthesis of* 1,3-Difluoro-2-nitrosobenzene **11**.A 250-mL single-neck flask was prepared, and 2,6-difluoroaniline (10, 3.22 g, 25.00 mmol) was weighed and added to the flask, followed by the addition of 50 mL of dichloromethane. The mixture was stirred at room temperature to accelerate dissolution. A solution of the solid oxidizing agent potassium peroxymonosulfate (Oxone, 19.28 g, 31.25 mmol) in 100 mL of water was prepared by sonication and then added dropwise to the single-neck flask. The reaction mixture was stirred at room temperature for an additional 3-5 hours, with the progress monitored by TLC until the reaction was complete. The reaction mixture was directly transferred to a separatory funnel and allowed to settle into layers. The lower organic phase was extracted, dried over anhydrous sodium sulfate, and filtered to obtain a dark green solution. After rotary evaporation under vacuum, the crude product 1,3-difluoro-2-nitrosobenzene **11**was obtained as a gray solid (3.396 g). This product can be directly used for the next step of the reaction.^1^H NMR (400 MHz, DMSO-d₆) δ 7.95 (ddd, *J* = 8.6, 6.2, 2.4 Hz, 1H), 7.47 (dd, *J* = 14.8, 6.0 Hz, 2H).

Synthesis of (E)-3,5-Difluoro-4-(2,6-difluorophenyldiazenyl)benzyl Alcohol **12**.A 250-mL two-neck flask was prepared and charged with compound 9 (2.70 g, 17.00 mmol) and compound 11 (2.92 g, 20.40 mmol). A mixed solvent (52 mL) composed of acetic acid, toluene, and trifluoroacetic acid in a ratio of 6:6:1 was added to the flask. The reaction was carried out under an argon atmosphere at room temperature, with the progress monitored by TLC. After approximately 37 hours, the stirring was stopped. For the work-up, 50 mL of saturated aqueous Na₂CO₃ solution was added to neutralize the reaction mixture. The mixture was then extracted with ethyl acetate, washed with water and saturated brine, dried over anhydrous magnesium sulfate, and purified by column chromatography using a heptane/ethyl acetate (2:1) solvent system. The pure product (E)-3,5-difluoro-4-(2,6-difluorophenyldiazenyl)benzyl alcohol **12** was obtained as an orange-yellow solid (0.56 g, yield 11.6%).^1^H NMR (400 MHz, CDCl₃) δ 7.36 (ddd, *J* = 14.3, 8.4, 5.9 Hz, 1H), 7.11-7.00 (m, 4H), 4.72 (s, 2H). ^19^F NMR (376 MHz, CDCl₃) δ -120.32 (d, *J* = 10.1 Hz), -121.38 (dd, *J* = 9.1, 5.9 Hz).

*Synthesis of* ***p*-F4ABAMI**.A 100-mL two-neck flask was prepared and charged with 2-amino-6-methyl nicotinic acid (0.340 g, 2.00 mmol), compound 12 (0.56 g, 1.972 mmol), and the catalyst 4-dimethylaminopyridine (0.055 g, 0.43 mmol). The reaction system was protected with an argon atmosphere. Anhydrous dichloromethane (25 mL) was slowly injected into the flask, and the mixture was stirred at room temperature to ensure homogeneity. Ethyl carbodiimide hydrochloride (0.347 g, 1.81 mmol) was dissolved in 6 mL of anhydrous dichloromethane and then slowly added to the reaction mixture. The reaction was stirred at room temperature for 28 hours until completion.The crude product was washed by stirring with 10 mL of saturated NaHCO₃ solution, extracted with ethyl acetate, and the organic layer was washed with water and saturated brine. The organic layer was dried over anhydrous magnesium sulfate, filtered through diatomaceous earth, and the filtrate was mixed with 300-400 mesh silica gel for column chromatography using pure dichloromethane as the eluent. The final product p-F4ABAMI was obtained as an orange solid (0.269 g, yield: 32.67%).

- ^1^H NMR (400 MHz, CDCl₃) δ 8.08 (d, *J* = 8.0 Hz, 1H), 7.38 (dq, *J* = 7.8, 5.6 Hz, 1H), 7.16 - 7.03 (m, 4H), 6.53 (d, *J* = 8.0 Hz, 1H), 5.32 (s, 2H), 2.42 (s, 3H).- ^19^F NMR (376 MHz, CDCl₃) δ -119.90 – -120.06 (m), -120.97 – -121.13 (m).- ^13^C NMR (101 MHz, CDCl₃) δ 165.41, 163.32, 158.29, 155.92, 155.88, 153.32, 153.28, 139.14, 130.56, 111.74, 111.50, 110.64, 110.40, 101.48, 63.46, 23.76.- ESI-HRMS: m/z for C₂₀H₁₄F₄N₄O₂ [M+H]⁺: 419.1131, found: 419.1121.

1. **Copies of NMR Spectra of Compounds**


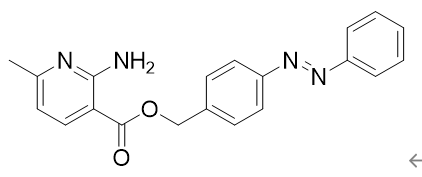

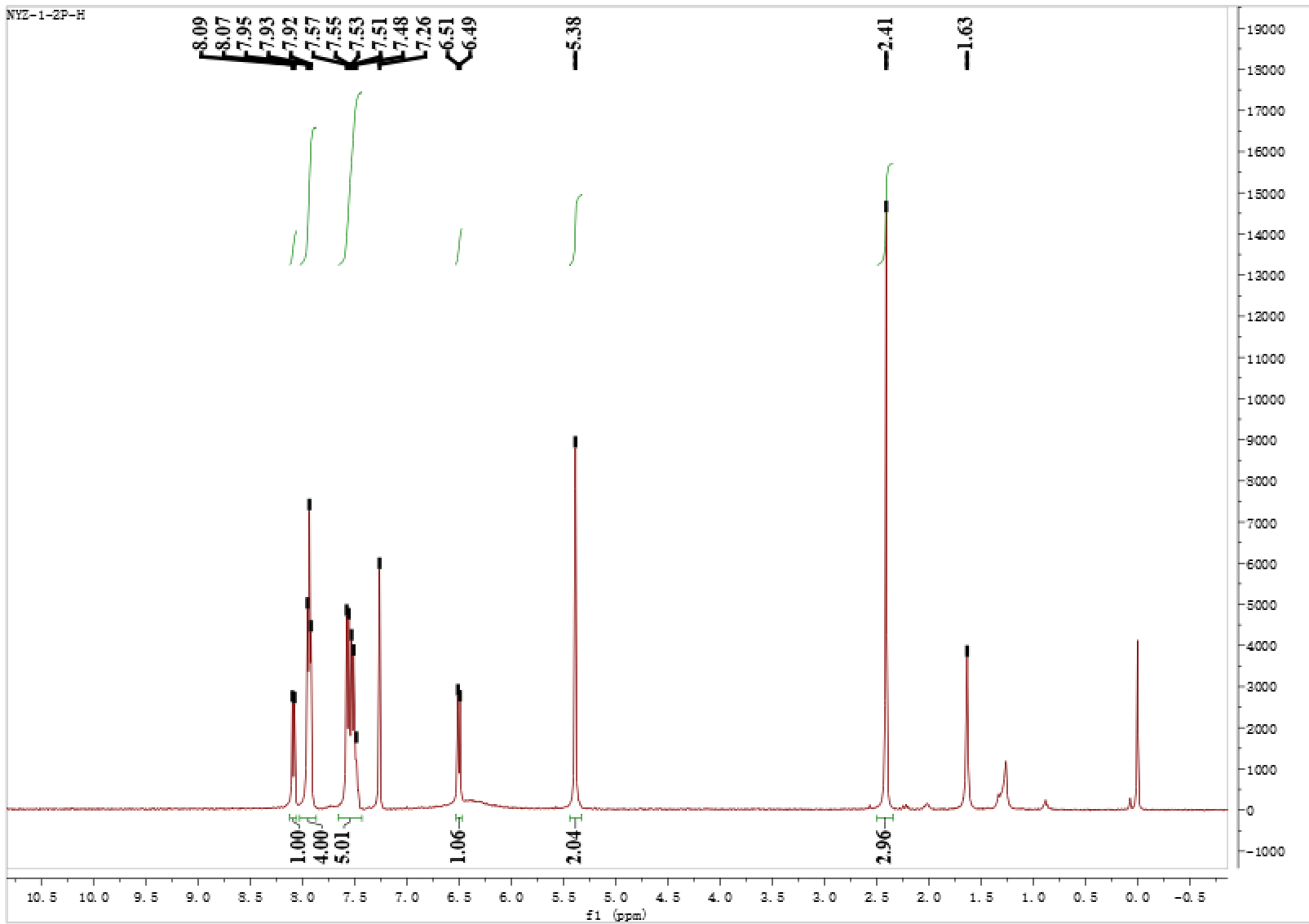


^1^H NMR spectrum of **ABAMI1**


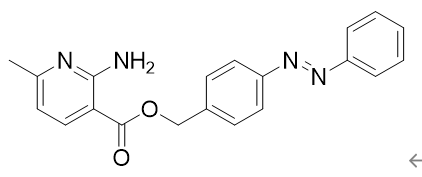

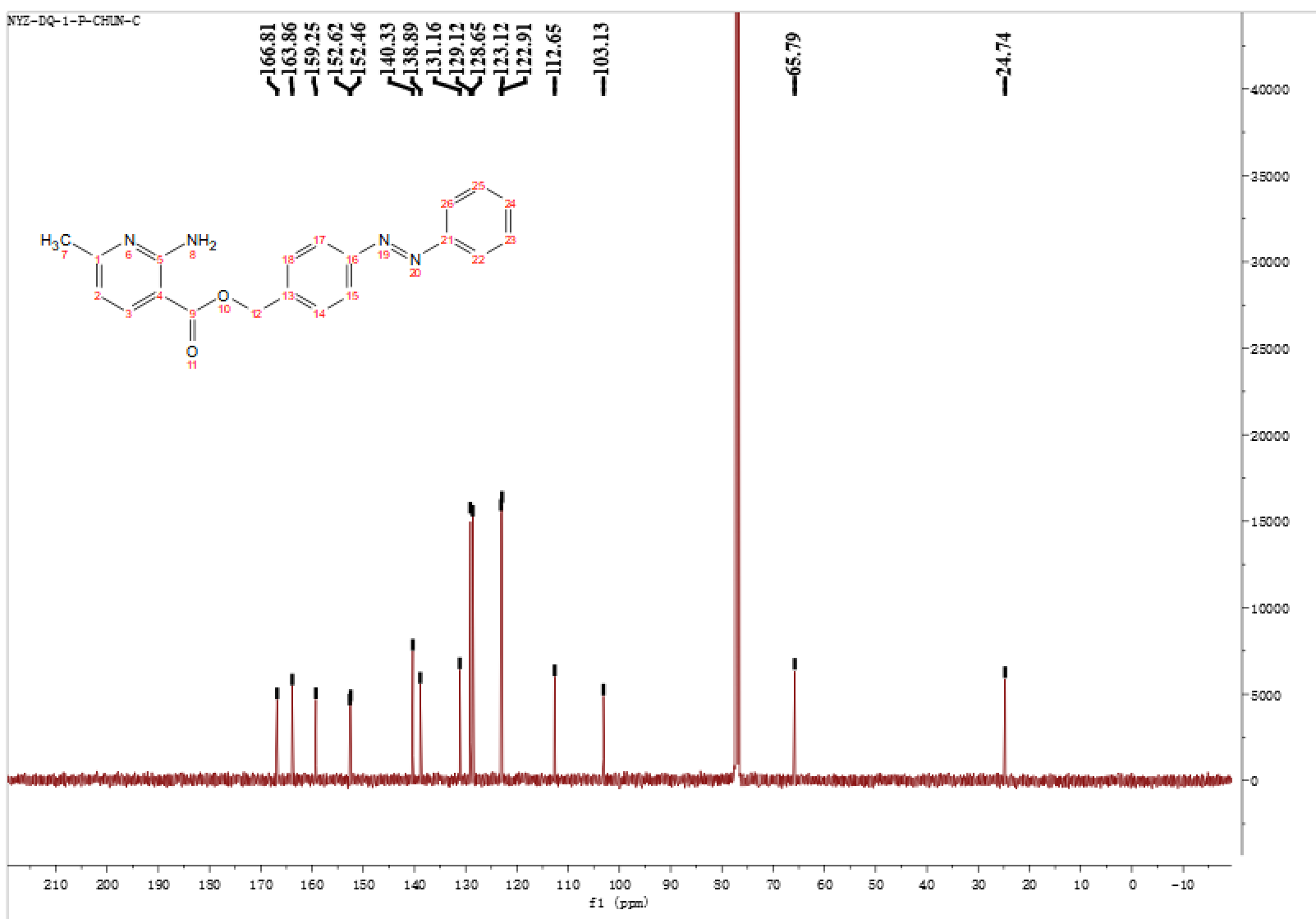


^13^C NMR spectrum of **ABAMI1**


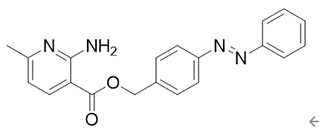

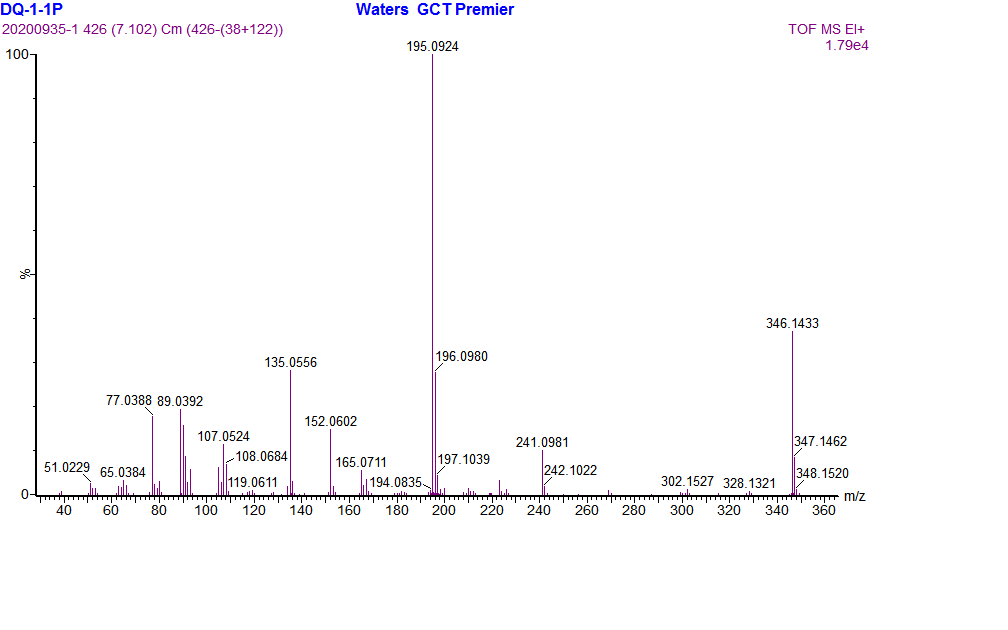


HRMS of **ABAMI1**


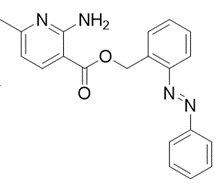

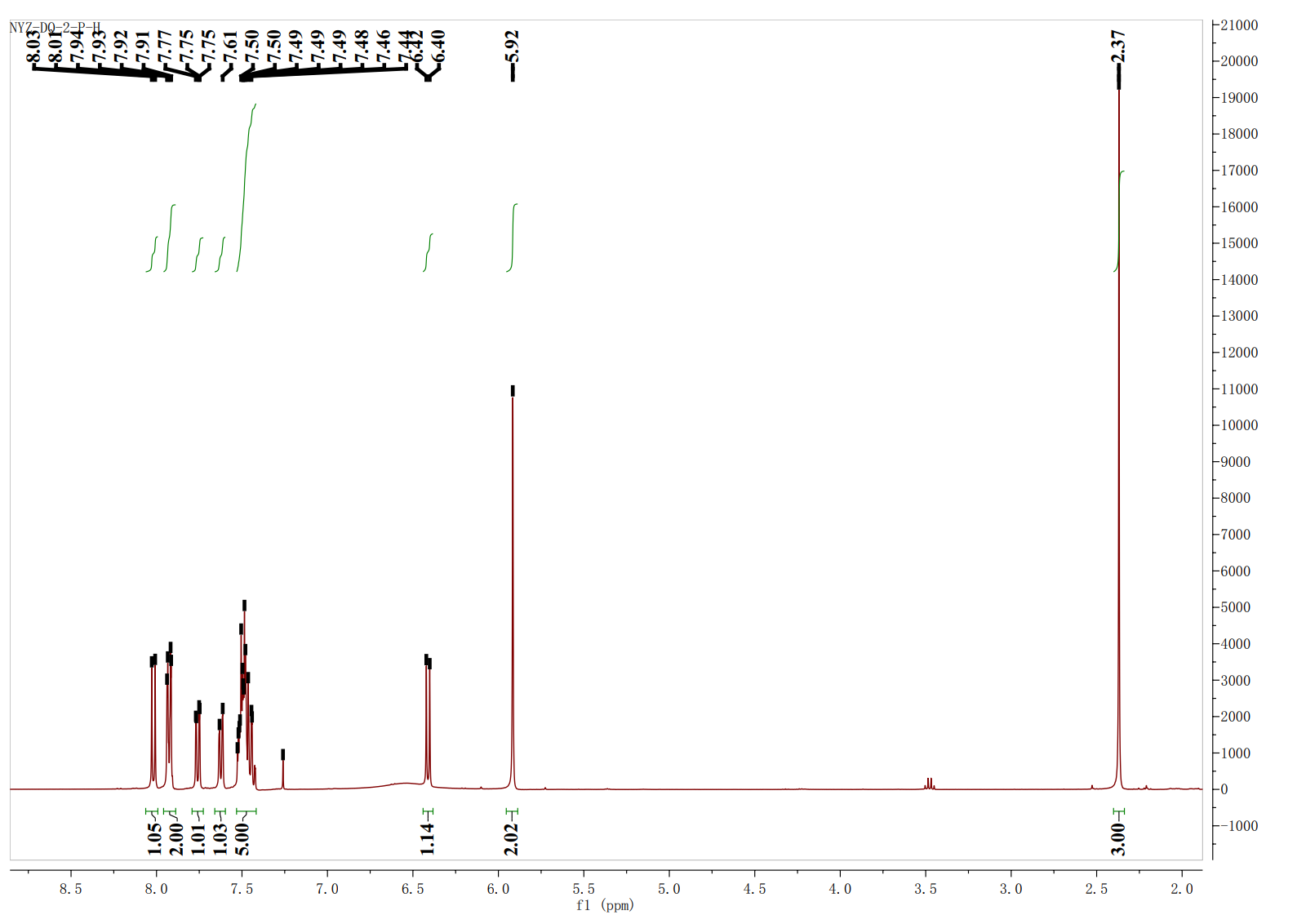


^1^H NMR spectrum of **ABAMI2**


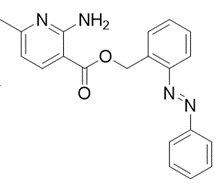

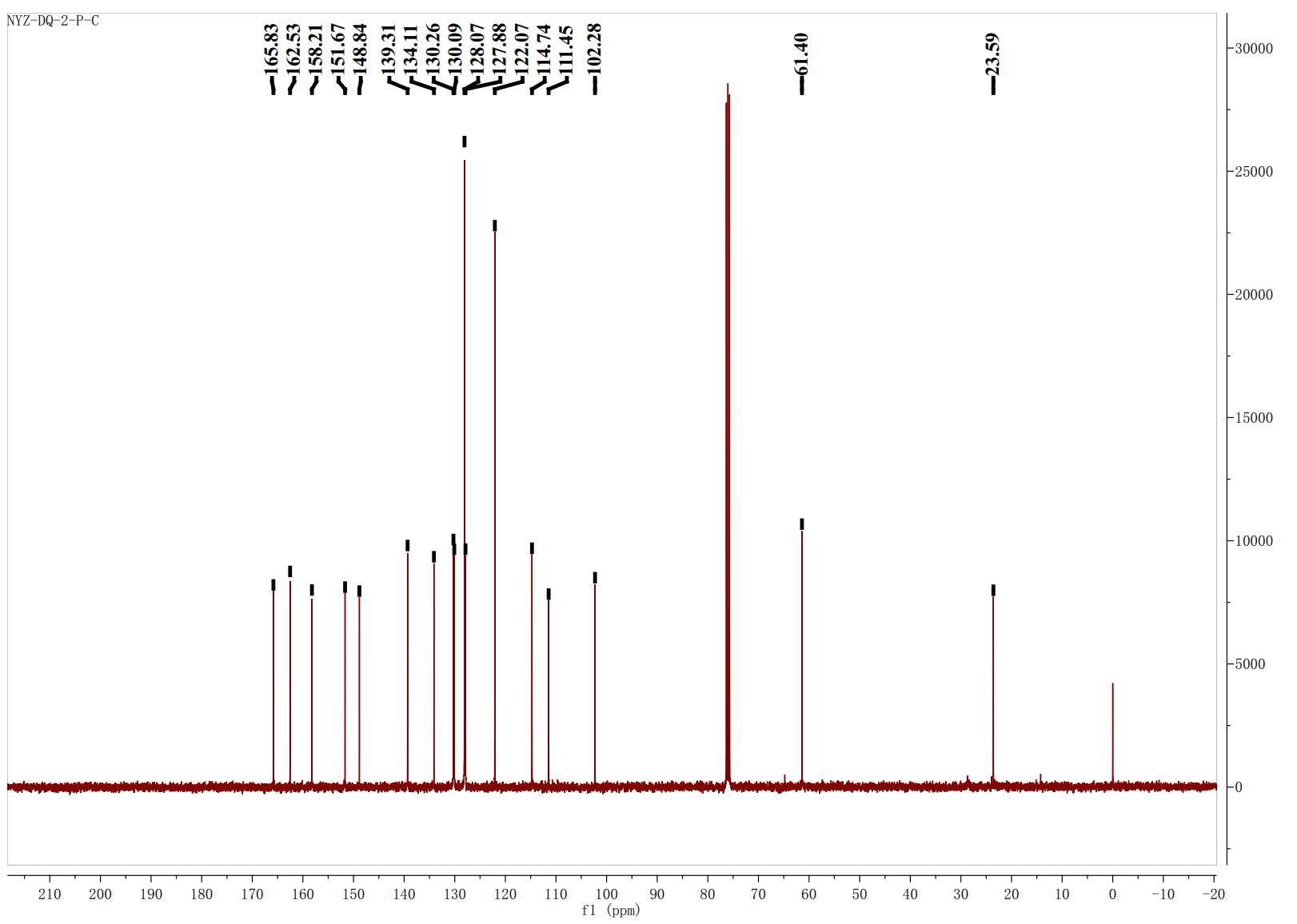


^13^C NMR spectrum of **ABAMI2**


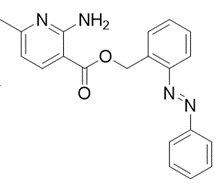


HRMS of **ABAMI2**


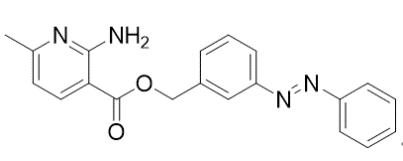

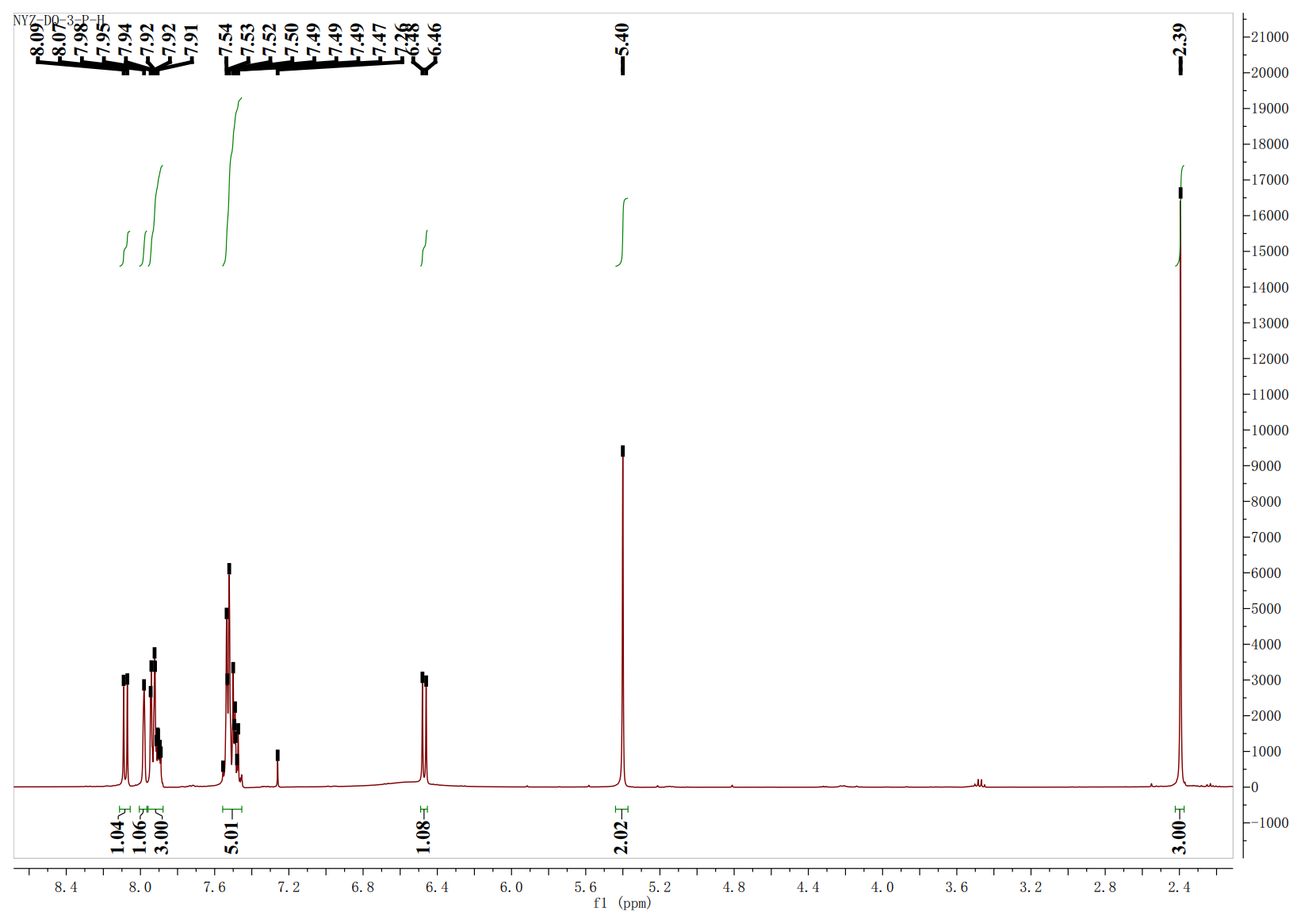


^1^H NMR spectrum of **ABAMI3**


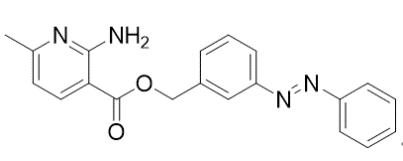

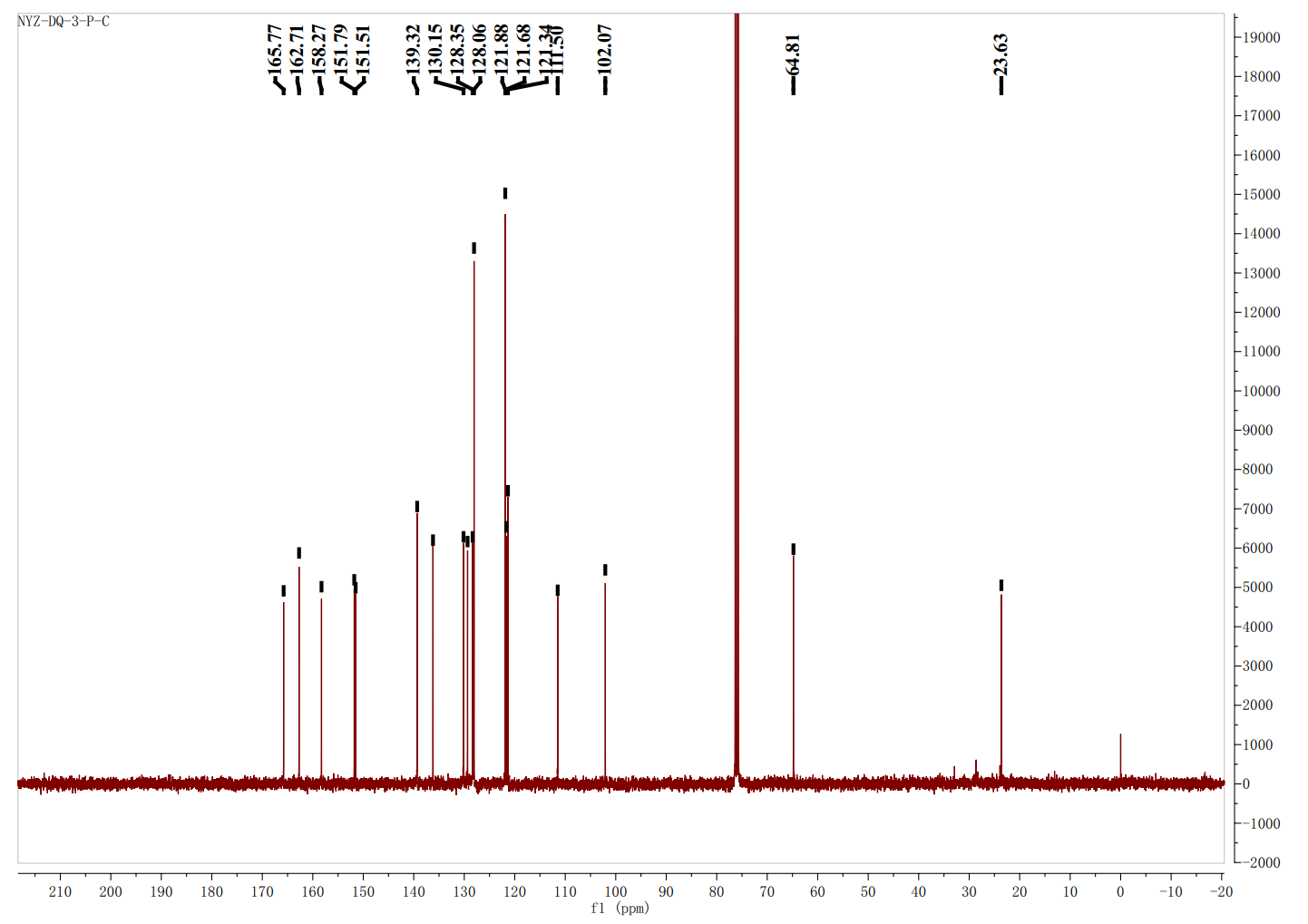


^13^C NMR spectrum of **ABAMI3**


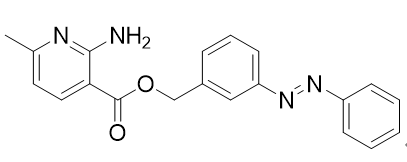

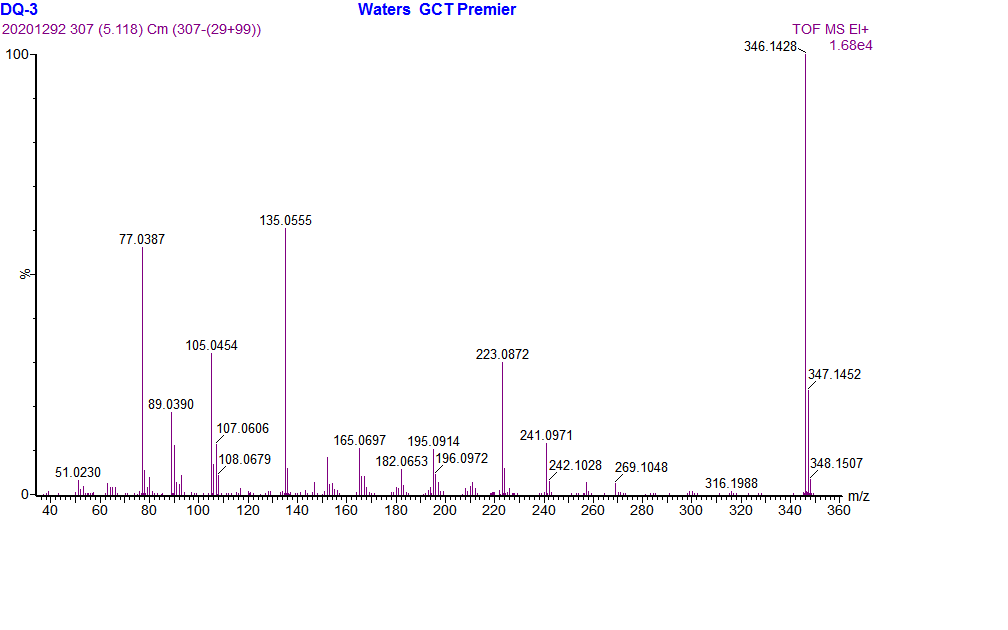


HRMS of **ABAMI3**

**
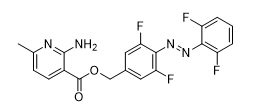

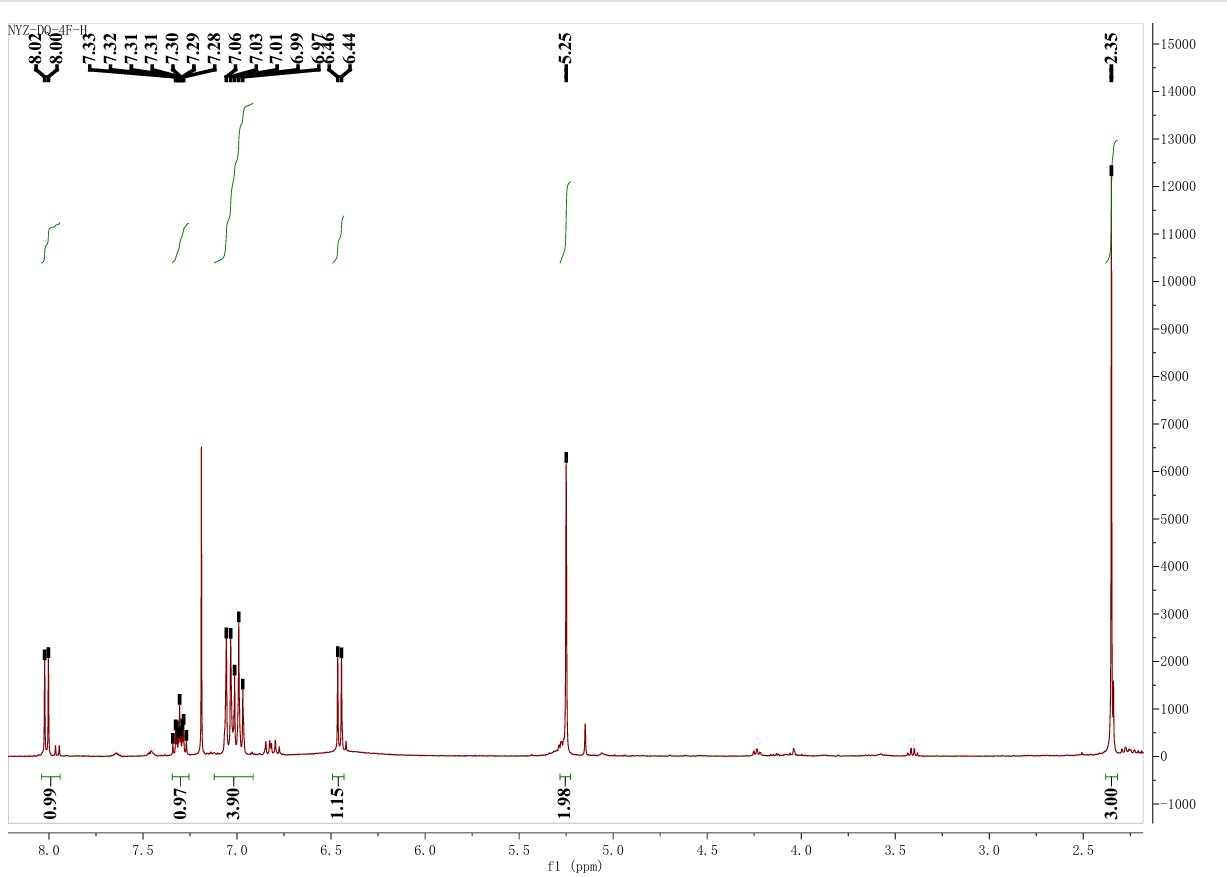
**^1^H NMR spectrum of ***p*-F_4_ABAMI** **
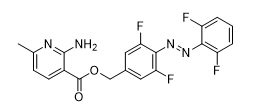

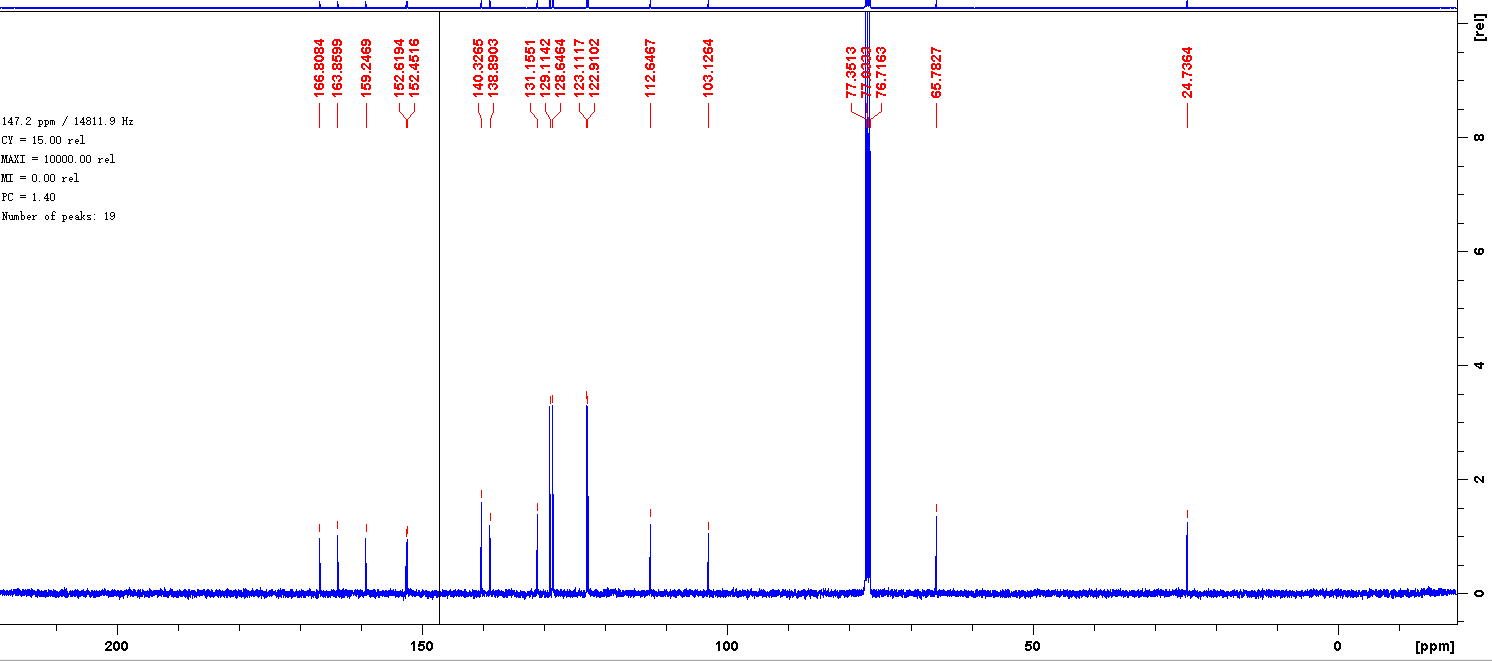
**^13^C NMR spectrum of ***p*-F_4_ABAMI**

**
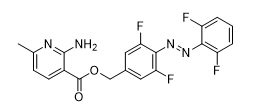

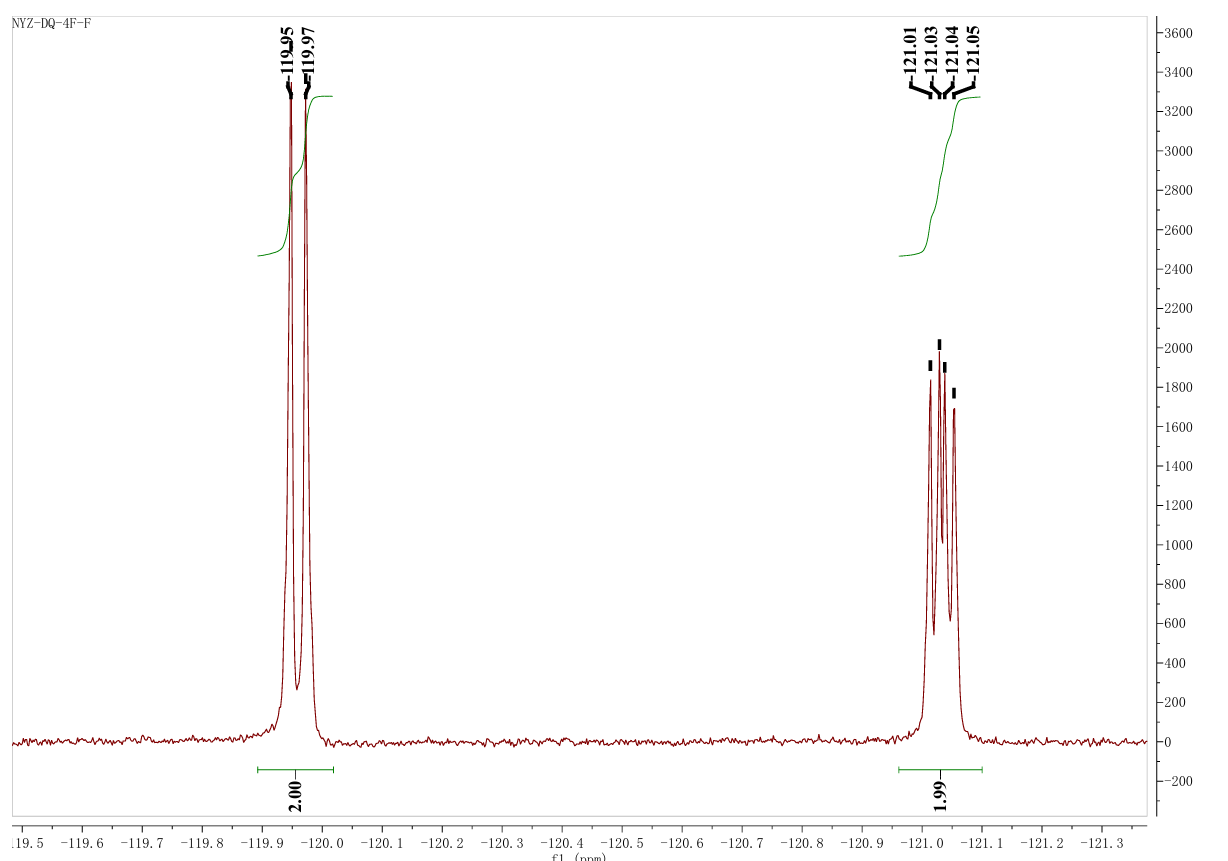
**

^19^F NMR spectrum of ***p*-F_4_ABAMI**

**
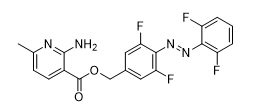

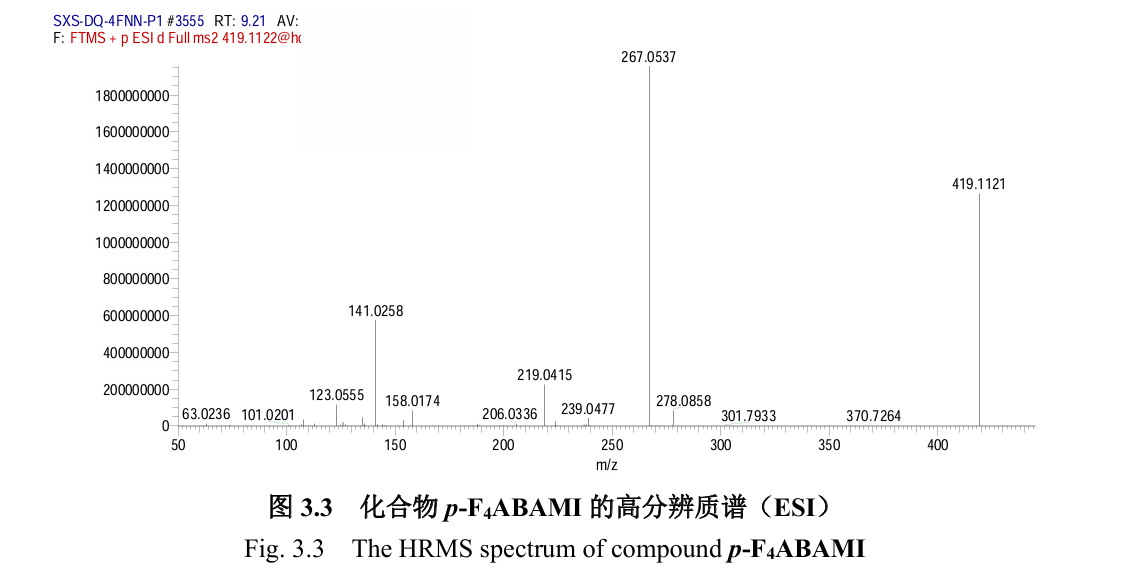
**

HRMS of ***p*-F_4_ABAMI**

1. **References**

[1] Xue T, Chao Z, Xu Q, et al. Azobenzene-Benzoylphenylureas as Photoswitchable Chitin Synthesis Inhibitors. *Organic & Biomolecular Chemistry*, 2017, 15, 3320-3323.

<https://doi.org/10.1039/C6OB02813F>.

[2] Yang W, Li M, Zhao J, et al. Wen Oligomycins a and C, major secondary metabolites isolated from the newly isolated strain Streptomyces diastaticus. *Folia Microbiol.* 2010, 55, 10-16. <https://doi.org/10.1007/s12223-010-0002-0>.

[3] Dongchao, Chen J, Tong M, et al. Inhibitory effects of methyl thujate on mycelial growth of Botrytis cinerea and possible mechanisms. Postharvest Biology and Technology, 2018, 142, 46-54. <https://doi.org/10.1016/j.postharvbio.2018.04.003>.
